# Modelling the impact of household size distribution on the transmission dynamics of COVID-19

**DOI:** 10.1098/rsif.2021.0036

**Published:** 2021-04-28

**Authors:** Pengyu Liu, Lisa McQuarrie, Yexuan Song, Caroline Colijn

**Affiliations:** ^1^ Department of Mathematics, Simon Fraser University, Burnaby, Canada V5A 1S6; ^2^ Department of Statistics and Actuarial Science, Simon Fraser University, Burnaby, Canada V5A 1S6

**Keywords:** COVID-19, infectious disease, household size, British Columbia

## Abstract

Under the implementation of non-pharmaceutical interventions such as social distancing and lockdowns, household transmission has been shown to be significant for COVID-19, posing challenges for reducing incidence in settings where people are asked to self-isolate at home and to spend increasing amounts of time at home due to distancing measures. Accordingly, characteristics of households in a region have been shown to relate to transmission heterogeneity of the virus. We introduce a discrete-time stochastic epidemiological model to examine the impact of the household size distribution in a region on the transmission dynamics. We choose parameters to reflect incidence in two health regions of the Greater Vancouver area in British Columbia and simulate the impact of distancing measures on transmission, with household size distribution the only different parameter between simulations for the two regions. Our result suggests that the dissimilarity in household size distribution alone can cause significant differences in incidence of the two regions, and the distributions drive distinct dynamics that match reported cases. Furthermore, our model suggests that offering individuals a place to isolate outside their household can speed the decline in cases, and does so more effectively where there are more larger households.

## 1. Introduction

The novel coronavirus disease (COVID-19) caused by severe acute respiratory syndrome coronavirus 2 (SARS-CoV-2) has created a global pandemic with over 50 million confirmed cases and more than one million deaths as of November 2020 [1]. In the absence of an effective cure and vaccine, various non-pharmaceutical interventions (NPIs), including hand hygiene, face masks, quarantine, isolation, contact tracing and social distancing, have been the primary practices for reducing the spread of the highly transmissible respiratory pathogen. Amid these interventions, stay-at-home policies and quarantine or isolation strategies may alter social interactions and hence the transmission dynamics of the virus, especially the transmission probabilities within and outside households [2,3].

It has been shown that the general secondary attack rate of COVID-19 to individuals within households is higher than that of severe acute respiratory syndrome (SARS) and Middle East respiratory syndrome (MERS) [4]. With higher contact rates within households under stay-at-home policies and strict lockdowns, investigating the connections between household characteristics and transmission dynamics of the virus could provide insights for designing interventions to prevent infection. A number of studies have found heterogeneity in the prevalence, hospitalization and mortality of COVID-19 related to demographic and ethnic differences among households and household size or household density. The findings indicate that individuals from ethnic minority backgrounds, especially South Asian and black individuals, are of higher risk related to COVID-19 [5–7], and household size may be associated with the risk of infection after implementing social distancing or stay-at-home policies [8]. A geospatial analysis has investigated the connections between socioeconomic factors in households and the prevalence of the disease, and indicates that lower educational attainment and higher household occupancy are among significant risk factors of infection [9]. An analysis based on detailed patient and contact tracing data has revealed that the average risk of transmission is positively associated with the closeness of social interactions, with highest risk within households, especially during lockdowns [10]. Other studies that consider household size show that controlling transmission within households is key to successfully bringing cases into a decline [11], and that small households are preferable for curbing an outbreak during a lockdown [12]. However, how different distributions of household size would affect transmission dynamics of the virus and the effectiveness of public health policies remains unknown.

The Greater Vancouver area incorporates two regional health authorities, dividing the metropolitan area into the Fraser Health (FH) region and the Vancouver Coastal Health (VCH) region. The numbers of COVID-19 cases in the two regions differ considerably, with approximately 18 000 total cases in FH and 8000 cases in VCH as of November 2020. The household size distributions of the two regions are also different. There are 1 695 150 individuals living in 631 135 private households in FH and 1 135 295 individuals living in 493 515 private households in VCH, according to Statistics Canada 2016 Census [13].

Household-stratified epidemic modelling incorporates population structure into epidemic models and studies the transmission heterogeneity caused by population behaviour [14]. Studies on household-stratified models have revealed that household transmissions (or local transmissions) have an amplification effect on the household-to-household reproduction number [15], and the household structure can have a significant influence on the performance of vaccination strategies [16]. To investigate the extent to which household size distributions in FH and VCH can affect the spread of the virus, we develop a discrete-time individual-based Markov-chain household-stratified SEIR model. We inform the model with data on the household size distribution in FH and VCH and with incidence data. We then analyse the impacts of household size distribution on the incidence of COVID-19 in FH and VCH, the probability of remaining uninfected for individuals living in households of different sizes, and the effectiveness of various isolation strategies.

## 2. Methods

### 2.1. Data

We obtained the household size distributions in the FH region and the VCH based on Statistics Canada 2016 Census [13], which includes 631 135 and 493 515 private households in FH and VCH, respectively. The data contain the size of private households in British Columbia and census subdivisions of British Columbia, listing the number of households with one to seven individuals and with at least eight individuals in each subdivision. We collect the data for all census subdivisions in FH and VCH regions and compute the total numbers and proportion of households of each size. COVID-19 data, including the daily incident cases in the Greater Vancouver area, are publicly available at British Columbia Centre for Disease Control (BCCDC) website [17], containing information of the dates and health regions of reported cases. We use the number of incident cases from each day between 3 March 2020 and 3 December 2020 in both FH and VCH.

### 2.2. Model description

We introduce an individual-based Markov chain susceptible-exposed-infectious-recovered (SEIR) model, which describes the time dynamics of susceptible (*S*), exposed (*E*), infectious (*I*) and recovered or deceased (*R*) individuals. For the sake of simplicity, we assume that households with at least eight individuals contain exactly eight individuals, and each simulated individual resides in a household of size one to eight individuals. The household size distribution in a simulation is a discrete probability distribution indicating the number of households of each size in a region. We also assume each individual spends a fraction *δ* of 24 h inside their household every day, and while an individual is in the community (outside the household), we assume the individual encountered *ρ* other individuals per day, not including the individuals in the same household. We suppose the transmission rate in the community per individual per day *β*_1_ is lower than the transmission rate within a household per individual per day *β*_2_, and that recovered individuals are immune to the virus.

We model transmission over *n* days in a region with *N* individuals. Let $\Omega ={\left\{S,E,I,R\right\}}^{N}$ be the state space, and $X_{i}=(X_{i}^{1},X_{i}^{2},\ldots ,X_{i}^{N})$ be an *N*-dimensional multivariate random variable representing the compartment each individual is in on day *i*. Specifically, $X_{i}^{k}$ denotes the compartment individual *k* is in on day *i*. We define a discrete-time Markov chain **X**_1_, **X**_2_, …, **X**_*n*_ in the following way.

2.1 $$\begin{array}{rl} & \mathrm{Pr}(X_{i+1}^{k}=E|X_{i}^{k}=S)=(1-\delta )\beta_{1}\rho ({\tilde{I}}_{i}^{k}/N)+\delta \beta_{2}{\hat{I}}_{i}^{k}\\ & \mathrm{Pr}(X_{i+1}^{k}=I|X_{i}^{k}=E)=\mu \\ \; & \mathrm{Pr}(X_{i+1}^{k}=R|X_{i}^{k}=I)=\nu \end{array}$$

**Figure 1.**
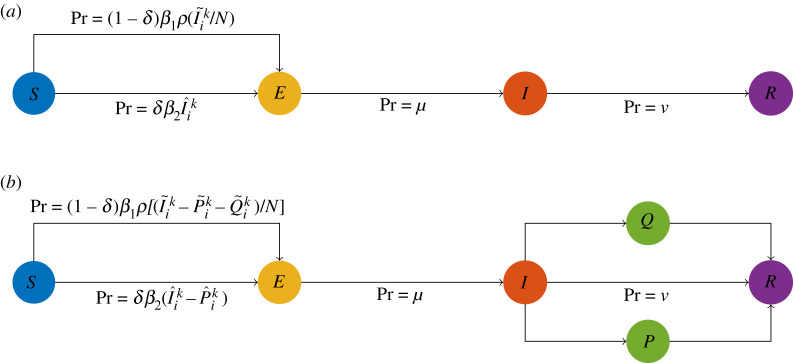
(*a*) The schematic diagram of the basic epidemiological model. (*b*) The schematic diagram of the adjusted epidemiological model with an isolation state, where *P* is the compartment of individuals practising separated isolation and *Q* is the compartment of individuals practising home isolation. The probabilities displayed in both diagrams are for each simulated individual.

This model has households and the community, such that each individual may contract the virus in either of these settings: from others in the same household, or from anyone in the community. The symbol ${\tilde{I}}_{i}^{k}$ denotes the number of individuals in compartment *I* on day *i* who are not from the same household as individual *k*, and the symbol ${\hat{I}}_{i}^{k}$ denotes the number of individuals in compartment *I* on day *i* who are from the same household as individual *k*. Note that equation (2.1) apply to every individual independently and the state of an individual affects the variables ${\tilde{I}}_{i}^{k}$ and ${\hat{I}}_{i}^{k}$ for all other individuals. This is the mechanism by which the household size distribution impacts transmission dynamics. The top schematic diagram in figure 1 provides a visual representation of the transmission dynamics governed by equation (2.1).

### 2.3. Scenarios

We study two scenarios. The first corresponds to the reported cases in the FH and VCH regions and investigates the potential impact of household size distribution on the incidence. The second is a hypothetical scenario examining the effectiveness of various isolation strategies under different household size distributions. For all simulations regarding the two major scenarios, we assign identical values to the universal parameters listed in table 1. We assign values for the parameters *β*_1_ and *β*_2_ based on the knowledge on household contacts and transmissions [18] and so that the basic reproduction number in the simulations is about 3 [19]. The values of *μ* and *ν* are chosen so that the incubation period falls in the range of 4.5–6.5 days [20,21] and the infectious period falls in the range of 5–20 days [22,23] in the simulations. The value for parameter *ρ* is chosen based on the study on social contacts [24]. All the parameters values are adjusted to reflect incidence in the Greater Vancouver area when necessary. For both scenarios, we generate simulated households randomly from the empirical household size distributions in FH and VCH so that the total number of simulated individuals in these households is *N* ≈ 500 000. We also assume that the population size *N* will not affect the transmission dynamics when the number of immune individuals is less than 1% of the simulated population.

**Table 1.** List of universal parameters and their values assigned for all scenarios analysed in this article.

parameter	definition	value
*β*_1_	transmission probability in the community per individual per day	0.011
*β*_2_	transmission probability within households per individual per day	0.09
*μ*	probability from compartment *E* to *I* per individual per day	0.15
*ν*	probability from compartment *I* to *R* per individual per day	0.071
*ρ*	number of individuals encountered in the community per individual per day	20

The first scenario has an initial date of 11 February 2020. We assume on the initial date, there exist 10 individuals in compartment *I* (selected uniformly at random from all simulated individuals in compartment *S*), 50 individuals in compartment *E* (selected uniformly at random from all simulated individuals in compartment *S* after selecting the individuals in compartment *I*), and no individual in compartment *R*. The length of a simulation for this scenario is 300 days, with 7 December 2020 as the last day. To model different phases of implementing stay-at-home policies and reopening, the parameter *δ* in this scenario takes on different values throughout a simulation. From the initial day to day 40 (21 March 2020), *δ* = 0.625 representing the baseline case when each individual approximately spends 15 h at home and 9 h in the community on average; see table 2 for detailed values chosen to reflect incidence in the Greater Vancouver area.

**Table 2.** The values of the parameter *δ* throughout a simulation for the first scenario.

date	value of *δ*
Day 1–40 (21 March 2020)	0.625
Day 40–47	0.625 to 0.925 (linearly)
Day 47–140 (29 June 2020)	0.925
Day 140–147	0.925 to 0.675 (linearly)
Day 147–210 (7 September 2020)	0.675
Day 210–217	0.675 to 0.875 (linearly)
Day 217–240 (7 October 2020)	0.875
Day 240–247	0.875 to 0.675 (linearly)
Day 247–300 (7 December 2020)	0.675

The second scenario is hypothetical and concerns isolation strategies. We adopt the same assumptions and initial conditions as in the first scenario. Moreover, we assign each individual a preference regarding how they would practise isolation if they are in compartment *I* when isolation is recommended. The possible preferences for an individual are: not practising isolation, practising isolation at home, and practising isolation at a separated place. We assume that individuals who prefer to not practise isolation can infect any other susceptible individual, individuals who prefer to practise isolation at home can infect only susceptible individuals in the same household, and individuals who prefer to isolate at a separated place cannot infect any susceptible individual. To model different isolation strategies, we modified the model according to (2.2), where ${\tilde{P}}_{i}^{k}$ and ${\tilde{Q}}_{i}^{k}$ denote the number of individuals who are not in the same household as individual *k*, and who are practising separated isolation and practising home isolation on day *i*, respectively. ${\hat{P}}_{i}^{k}$ denotes the number of individuals who are in the same household as individual *k* and who are practising separated isolation on day *i*. For a visual representation of this model, see the bottom schematic diagram in figure 1.

2.2 Pr(Xi+1k=E|Xik=S)=(1−δ)β1ρ[(I~ik−P~ik−Q~ik)/N]+δβ2(I^ik−P^ik)Pr(Xi+1k=I|Xik=E)=μ   Pr(Xi+1k=R|Xik=I)=ν

In this scenario, the parameter *δ* = 0.675 remains constant for all simulated days and the length of a simulation is 200 days. From the initial day to day 50, infected individuals are not recommended to isolate, meaning no individual practises any type of isolation. Starting from day 51 to the end of a simulation, the simulated individuals practise isolation with respect to their preferences.

To compare the effectiveness of different isolation strategies in regions with different household size distributions, we designed four isolation scenarios listed in table 3. The only parameters that vary between the isolation scenarios are the household size distribution (between FH and VCH) and the distribution of the isolation preferences over the simulated individuals. We keep all other parameter values in each of the simulations identical for all isolation scenarios. The isolation scenario FH-H (home isolation) uses the household size distribution in FH. 55% of the simulated individuals would practise isolation at home when they are in compartment *I* while the other 45% simulated individuals would not practise isolation. Similarly, the isolation scenario FH-S (separated isolation) also uses the household size distribution in FH, with the difference that 55% of the simulated individuals would practise isolation at a separated place and the remaining 45% would not practise isolation. The distribution of isolation preferences over the simulated individuals in isolation scenarios VCH-H and VCH-S is the same as in scenarios FH-H and FH-S, respectively, but these isolation scenarios are with the household size distribution in VCH.

**Table 3.** List of isolation scenarios, corresponding isolation preferences and household size distribution in use.

isolation scenario	no isolation	home isolation	separated isolation	household distribution
FH-H	45%	55%	0%	FH
FH-S	45%	0%	55%	FH
VCH-H	45%	55%	0%	VCH
VCH-S	45%	0%	55%	VCH

### 2.4. Probability of remaining uninfected

We use survival analysis techniques to analyse the probability of an individual becoming infected on each simulated day. We apply a Kaplan–Meier estimator [25] to estimate the probability of remaining not infected for individuals in households of different sizes. Note that the event of interest here is the infection of an individual, so ‘survival’ means remaining uninfected. Let *N*^*k*^ be the number of individuals in households of size *k*, and $I_{i}^{k}$ be the number of incident cases from households of size *k*, on day *i*. The survival function *L*^*k*^(*t*), indicating the probability of remaining uninfected for individuals from households of size *k* on day *t*, is defined by the standard formula: $L^{k}(t)=\prod_{i=1}^{t}[1-I_{i}^{k}/(N^{k}-\sum_{\;j=1}^{i}I_{j}^{k})]$.

## 3. Results

### 3.1. Distribution comparison

**Figure 2.**
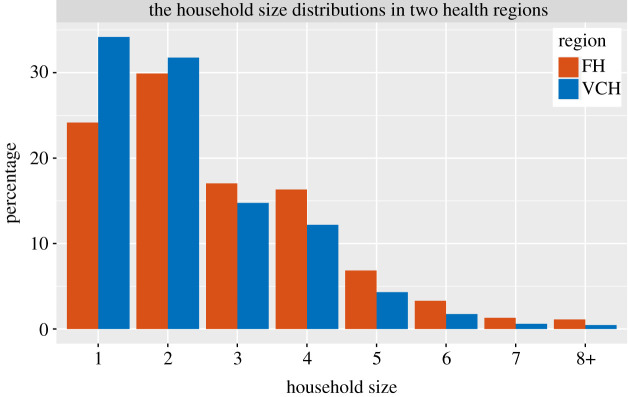
The household size distributions in the Fraser Health region and the Vancouver Coastal Health region.

The household size distributions in the two health regions are different; the average household size is 2.68 in FH and is 2.31 in VCH. There are more large households and fewer single-individual households in FH; see figure 2. We compare the distributions by Pearson’s χ^2^ test [26], which rejects the null hypothesis that the household sizes in FH and VCH originate from populations with the same distribution with a *p*-value 2.2 × 10^−16^. This indicates that there are differences between the household size distributions in the two health regions.

### 3.2. Impact on incidence

**Figure 3.**
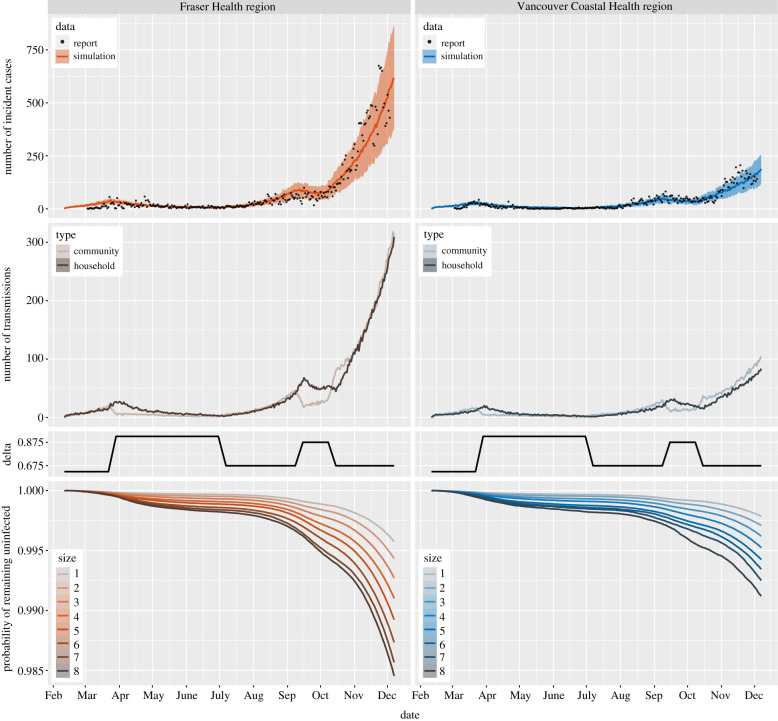
Top: the number of incident cases from simulation (mean curves with 10–90 percentile range bands) and reported data (points) in the two regions. Middle: the first row displays the number of community and household transmissions from simulations in the two regions; the second row depicts the values of parameter *δ* described in table 2. Bottom: the survival curves for individuals in households of different sizes from simulations in the two regions. All curves in the figure reflect mean values over 100 runs for each of the simulations. Model parameters are the same in the two regions, except for the household size distribution.

We apply the model to analyse the impact of household size distribution on the incidence of COVID-19 in FH and VCH. We set the simulations for FH and VCH with the same initial values and parameters described in §2.3, except for the household size distribution, which is initialized to match the observed household size distribution in each health region. We repeat the simulation for each health region 100 times; figure 3 displays the results. The top two panels show the number of incident cases from simulations and reported data. Note that the simulation results match the reported cases and the only parameter that differs between simulations for the left and right panels is the household size distribution. While reported cases likely do not represent all cases, for simplicity, we assume a constant ascertainment fraction. These results indicate that the difference between the household size distributions in FH and VCH can lead to a substantial difference in COVID-19 incidence, suggesting that the household size distribution may be a factor causing the heterogeneity in the number of COVID-19 cases in FH and VCH.

We also plot the number of transmissions in the community and within households (middle two panels of figure 3). The results show that under the settings described in §2.3, the number of transmissions in the community and within households are similar. When stay-at-home policies are implemented, the number of community transmissions decreases but the number of household transmissions keeps increasing for several days.

### 3.3. Probability of remaining uninfected

The bottom panels of figure 3 show the mean probability of remaining uninfected up to day *n*, over 100 runs of the simulations, for both health regions. Note that the probabilities in these plots depend on the total population size in the model (here 500 000 individuals). Without knowledge of the percentage of infected individuals who are tested or the true number of infected individuals at the beginning of the simulation, it is not possible to relate the model’s probability of remaining uninfected to the true prevalence.

We find that individuals living in larger households have lower probability of remaining uninfected, and for each household size, the individuals living in FH have lower probability of remaining uninfected than individuals living in VCH, especially near the end of the simulations, due to the difference in prevalence of the two health regions. Moreover, under stay-at-home policies and social distancing measures, the probability of remaining uninfected for individuals living in large households decreases more substantially than for individuals living in small households.

### 3.4. Isolation effectiveness

**Figure 4.**
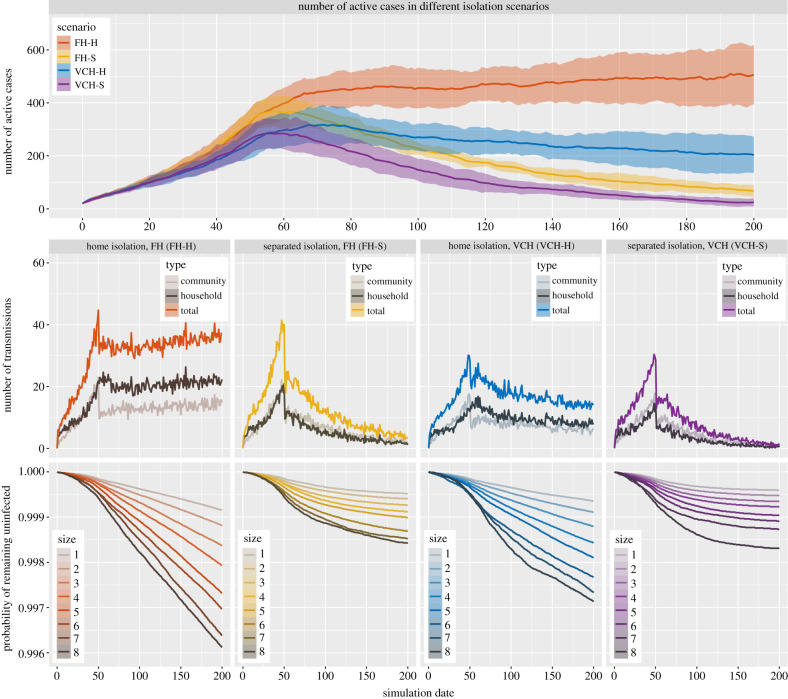
Results of simulations for isolation scenarios where 55% of the simulated individuals practise home or separated isolation. Top: the number of active cases (mean with 10–90 percentile range bands) in each of the isolation scenarios. Middle: the number of transmissions of different types in each of the isolation scenarios. Bottom: the survival curves for individuals in households of different sizes in each of the isolation scenarios. All curves in the figure reflect mean values over 100 runs of the simulation for each of the isolation scenarios and the settings only differ in household size distribution and the individuals’ isolation preferences. The curves are organized so that warm colours (red and yellow) represent scenarios in FH and cold colours (blue and purple) represent scenarios in VCH.

Figure 4 shows the differences in incidence under different isolation strategies, based on 100 runs of the simulations for the second scenario described in §2.3. The top panel of figure 4 shows the number of active cases in each of the four isolation scenarios. The results suggest that a proportion of individuals isolating at a separated place can result in more rapid decreases in cases than the same proportion of individuals isolating at home. Interestingly, with the same settings, 55% of the simulated individuals isolating at home can bring the cases into a decline under the household size distribution in VCH, while the number of cases continues to increase at a moderate rate under the household size distribution in FH. Comparing the bottom panels of figure 4 indicates home isolation poses a lower probability of remaining uninfected than separated isolation for individuals in households of all sizes including individuals living by themselves. Moreover, 55% of individuals isolating at home under our settings would reduce the growth of both community and household transmissions, though it makes household transmission more prominent than community transmission; see the middle panels of figure 4.

Unless there is such widespread testing in place that individuals know they are infectious very early in their infection, an individual who becomes infectious would not practise isolation immediately, but would begin after a period of time when the individual receives a positive test or develops symptoms. We add this period of time to our simulations described in electronic supplementary material, figure S1 shows the results. We find that home isolation after this delay is naturally less effective compared to our hypothetical experiment, and it is even more so for separated isolation in the two regions. However, it is still the case that separated isolation can result in more rapid decreases in cases than isolating individuals at home. Moreover, offering a separated isolation place for individuals who live in households with four or more individuals when 55% of the individuals practise home isolation under the household size distribution in FH can bring the cases into a decline and increase the probability of remaining uninfected for all individuals.

We also alter the proportion of simulated individuals practising either home or separated isolation. Electronic supplementary material, figures S2 and S3 display the results for the analogous four scenarios with 75% and 25%, respectively, of the simulated individuals practising either home or separated isolation and the rest of the individuals not practising isolation. The results suggest that home isolation can bring cases into a decline if 75% are able to practise isolation, under either the household size distribution in FH or VCH, though in this case individuals living in larger household are of lower probability of remaining uninfected compared with separated isolation. Conversely, if not enough individuals practise isolation (here only 25%), even though some individuals practise the strict separated isolation, the intervention is insufficient to result in declining cases; see electronic supplementary material, figure S3.

## 4. Discussion

We have developed a stochastic model and used it to investigate the impacts of household size distribution and home versus separated isolation on the incidence of COVID-19. We have chosen an individual-based stochastic model over a deterministic model because the number of infections is small compared to the number of households in the scenarios. The model has been designed to be as simple as possible, with only the essential components to discover how the distributions of household size would affect transmission dynamics. Our model does not simulate the entire population of the health regions, limiting our ability to compare the absolute probability of infection. Our model also does not include an explicit simulation of contacts within and between schools, retail and social settings and workplaces, or finer geographical variation within FH and VCH regions, and indeed the data to support modelling of these complex contact structures at a high level of temporal resolution is generally not available. Some further improvements to make the model more realistic include introducing gamma distributions for time an individual remains exposed or infectious.

We have found that under parameters reflecting COVID-19 transmission in British Columbia, the difference in household size distribution alone can account for the distinct transmission dynamics in the two health regions we have studied. We also find that in the context of directives to stay home, and to self-isolate at home if ill, an individual’s household size has a high impact on their probability of remaining uninfected. These results suggest that the household size distribution may be a key factor of transmission heterogeneity for COVID-19. Our results also show that an isolation strategy can be successful under one distribution of household size at controlling the spread of the virus but less effective under a different household size distribution, indicating that uniform policies for regions with different demographic characteristics may not be optimal. Jurisdictions with many larger households would benefit more from policies offering self-isolation at a separated place than jurisdictions with predominantly smaller households. Furthermore, at rates of transmission that are comparable to those in the Greater Vancouver area, which are likely relatively near the epidemic threshold at the time of writing, this difference could even be enough to bring COVID-19 cases into a decline.

There are a number of sources of disparity and inequity that have been found to be connected to COVID-19 risk, including the physical size of households (and therefore the density of contact), occupation [27], age [4,28], ethnicity [5,7], income [9,29] and comorbidities. These intersect: larger households may have several members who are essential workers who must work outside the home, lower-income employment is less likely to allow working from home [30] and households with more members may also be more crowded. Furthermore, evidence indicates that those living in neighbourhoods with high proportions of essential workers had higher rates of COVID-19 infection and death than those living in other neighbourhoods [31]. The intersection of these inequalities lends further urgency to the need to develop targeted support, including offering a separate place to isolate.

## Data accessibility

Source data about households are from Statistics Canada 2016 Census and are available at https://www12.statcan.gc.ca/census-recensement/2016/dp-pd/index-eng.cfm British Columbia COVID-19 case data are from BCCDC and are available at http://www.bccdc.ca/health-info/diseases-conditions/covid-19/data. The data and code for simulations in this article are available at https://github.com/pliumath/covid19-householdsize.
